# Impact-Free Measurement of Microtubule Rotations on Kinesin and Cytoplasmic-Dynein Coated Surfaces

**DOI:** 10.1371/journal.pone.0136920

**Published:** 2015-09-14

**Authors:** Aniruddha Mitra, Felix Ruhnow, Bert Nitzsche, Stefan Diez

**Affiliations:** 1 B CUBE—Center for Molecular Bioengineering, Technische Universität Dresden, Dresden, Germany; 2 Max Plank Institute of Molecular Cell Biology and Genetics, Dresden, Germany; Politecnico di Milano, ITALY

## Abstract

Knowledge about the three-dimensional stepping of motor proteins on the surface of microtubules (MTs) as well as the torsional components in their power strokes can be inferred from longitudinal MT rotations in gliding motility assays. In previous studies, optical detection of these rotations relied on the tracking of rather large optical probes present on the outer MT surface. However, these probes may act as obstacles for motor stepping and may prevent the unhindered rotation of the gliding MTs. To overcome these limitations, we devised a novel, impact-free method to detect MT rotations based on fluorescent speckles within the MT structure in combination with fluorescence-interference contrast microscopy. We (i) confirmed the rotational pitches of MTs gliding on surfaces coated by kinesin-1 and kinesin-8 motors, (ii) demonstrated the superiority of our method over previous approaches on kinesin-8 coated surfaces at low ATP concentration, and (iii) identified MT rotations driven by *mammalian* cytoplasmic dynein, indicating that during collective motion cytoplasmic dynein side-steps with a bias in one direction. Our novel method is easy to implement on any state-of-the-art fluorescence microscope and allows for high-throughput experiments.

## Introduction

Motor proteins from the kinesin- and dynein-superfamilies fulfill essential mechano-chemical functions in eukaryotic cells. Recently, the one-dimensional motion of these motors along microtubules (MTs) has been studied in great detail using *in vitro* stepping motility assays, for example by tracking single fluorescently labeled motors [1,2] as well as motors coupled to microbeads [3–5], quantum dots (QDots) [6–8] or DNA origami [9,10]. However, MTs are three-dimensional, cylindrical structures (diameter of 25nm), which consist of about 13 adjacent protofilaments forming a parallel array of tracks. While some kinds of processive motors follow the axes of individual protofilaments, others take stochastic off-axis steps potentially with or without bias in one specific direction. One simple experimental method to distinguish between these modes of movement is based on *in vitro* gliding motility assays where MTs glide over motor-coated surfaces in the presence of ATP. Functionalizing the MTs with optical markers, which have the potential to report on longitudinal rotations of individual MTs during forward movement, then allows inference of the helicity by which the motors walk on the MT surface. Using this strategy, it has previously been shown that kinesin-1 (conventional kinesin) follows the axis of individual protofilaments [11,12], while kinesin-8 (Kip3) switches protofilaments with a bias to the left [13]. Furthermore, non-processive motor proteins like kinesin-14 [14], axonemal dynein [15,16] and single monomeric kinesin-1 [17] have been shown to exhibit an inherent torsional component in their power strokes.

In early experiments, inference of MT rotation relied on supercoiling of partially stuck MTs [16,18], deflection of short side arms [12,17] or curvatures at the ends of deformed MTs [14,15]. In more recent studies, QDots coupled to MTs were used as optical probes in combination with three-dimensional tracking techniques based on dual-focus imaging [17,19] or fluorescence-interference contrast (FLIC) microscopy [12,13]. While QDots (diameter of about 20-30nm) are significantly smaller than previously used structures (e.g. MT side arms with lengths of about 1 μm) it cannot be ruled out that their presence limits the accuracy of the rotation measurements, especially in cases where torque generation by the motor proteins is weak.

**Fig 1:**
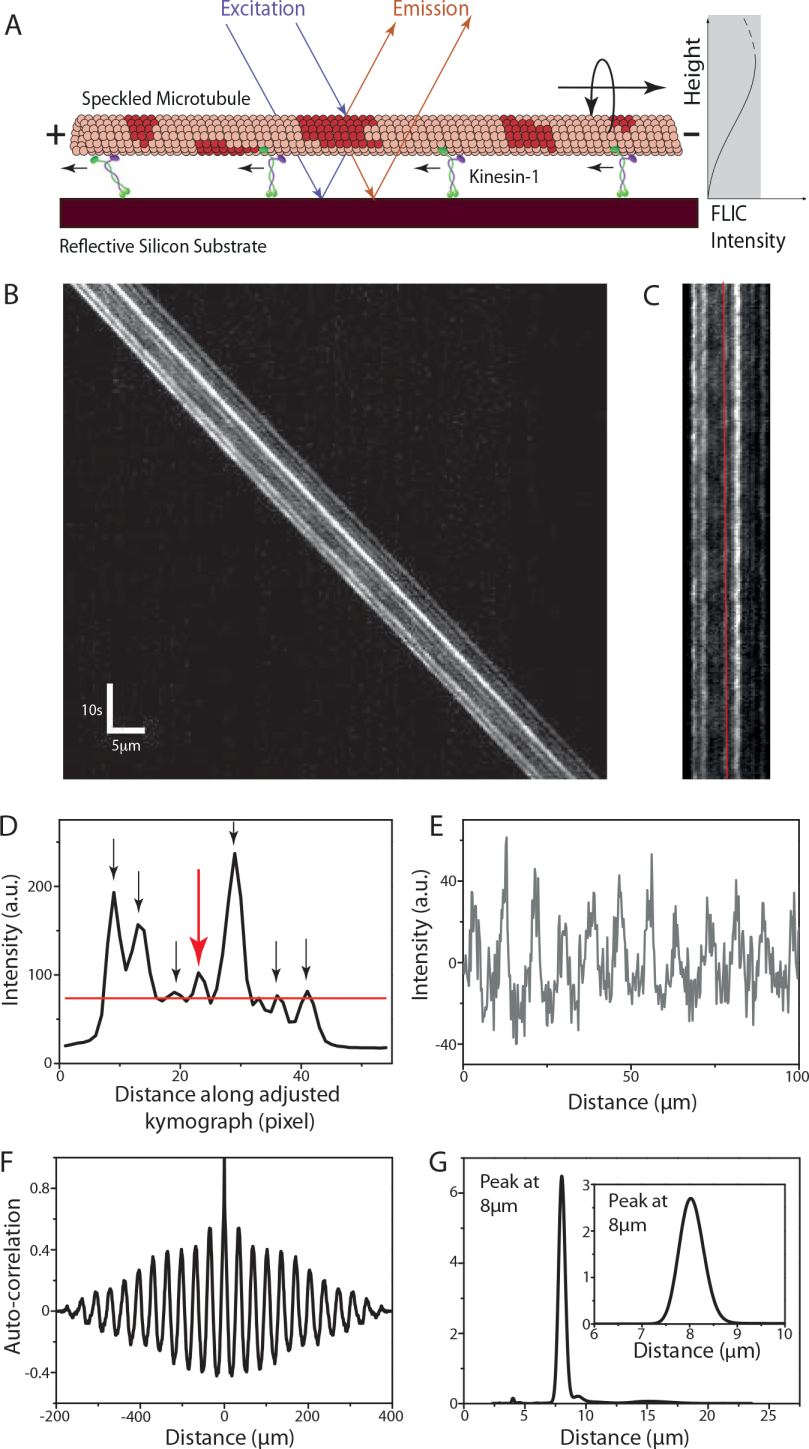
Analysis of the rotational pitch of a speckled MT (S-MT) gliding on kinesin-1. **(A)** S-MTs glide on a reflective silicon substrate coated with kinesin-1 motor proteins. Due to fluorescence interference contrast (FLIC), the recorded intensities of the speckles vary as a function of height above the surface. Torsional motion of gliding S-MTs leads to periodic variations in the intensity of each speckle providing information about the rotational pitch. **(B)** Typical kymograph (space-time intensity plot) of a gliding S-MT. Each individual speckle shows a periodic intensity variation. **(C)** After background correction, the kymograph is straightened by shifting every time frame by the distance the S-MT moved. **(D)** Averaged intensity profile along the straightened kymograph from Fig 1C. The red line shows the mean intensity and only speckles with peaks above this line (indicated by the arrows) are analyzed. **(E)** Relative intensity profile (centered around zero) over distance for the speckle along the red line in Fig 1C and indicated by the red arrow in Fig 1D. This profile is obtained by translating the intensity profile over time to distance in order to account for velocity variations of the gliding S-MT. **(F)** Auto-correlation for the intensity plot in Fig 1E. **(G)** Combined power spectral density (PSD) curve for the seven speckles selected in Fig 1D (see S1 Fig for details on the other speckles), with peak at about 8μm. **Inset:** Power spectrum of the auto-correlation data in Fig 1F, with peak at about 8μm.

To overcome this limitation, we devised a novel, impact-free method to measure the longitudinal rotations of gliding MTs without the necessity of large markers external to the structure of MTs. In particular, we reconstituted fluorescent MTs with rhodamine speckles acting as optical probes. We detected the rotational movements of individual speckles during MT movement on a reflective surface by FLIC microscopy, where a modulation in the height of a fluorescent probe above the surface is converted into a modulation of the detected fluorescence intensity [20]. Periodic intensity variations in the individual speckles then report on the rotational pitch of the gliding MTs and thus the helical movement of motors on the MTs (see Fig 1).

## Methods

### Protein expression and purification

Histidine-tagged full-length *Drosophila melanogaster* kinesin-1 and histidine-eGFP tagged *Saccharomyces cerevisiae* kinesin-8 (Kip3-eGFP-6xHis, in the text referred to as Kip3-eGFP) were expressed and purified using established protocols as described previously [21,22]. *Porcine* cytoplasmic dynein and *porcine* tubulin were purified from *porcine* brain (Vorwerk Podemus, Dresden, Germany) using established protocols as described previously [23,24].

### Speckled MTs

Guanylyl-(α,β)-methylene-diphosphonate (GMP-CPP) speckled MTs (S-MTs) were grown in a two-step process. 25μl of BRB80 solution (80mM Pipes [Sigma], pH 6.9, with KOH [VWR], 1mM EGTA [Sigma], 1mM MgCl_2_ [VWR]) supplemented by a high concentration (12μM) of *porcine* tubulin (S-MTs: 99.44% unlabeled and 0.56% rhodamine-labeled; biotinylated S-MTs [B-S-MTs]: 94.44% unlabeled, 5% unlabeled biotinylated (Cytoskeleton Inc., Denver, CO) and 0.56% rhodamine-labeled), 1 mM GMP-CPP (Jena Bioscience, Jena, Germany) and 4mM MgCl_2_ were incubated on ice for 5min and then for 20-30min at 37°C to grow a large number of short, dimly-labeled MT seeds. In parallel, an elongation mix consisting of a 100μl BRB80 solution supplemented by a low concentration (0.8μM) of *porcine* tubulin (96.67% unlabeled and 3.33% rhodamine-labeled), 1mM GMP-CPP and 4mM MgCl_2_ was heated to 37°C for 30s (after a 5min incubation on ice). Afterwards, 10μl of the dimly-labeled seed solution (containing S-MT or B-S-MT seeds) was added to the elongation mix (resulting total tubulin concentration ≈ 2μM) and incubated at 37°C for 3 hours. During this incubation period, bright extensions grew off the dim seeds and formed the speckles in the resulting S-MT and B-S-MT lattices after concurrent MT end-to-end annealing. Assembled S-MTs and B-S-MTs were centrifuged using a Beckman airfuge (Beckman, Brea, CA) at 100,000g for 5min. The pellet was resuspended in a volume of 50–100μl BRB80T (BRB80 supplemented with 10μM Taxol [Sigma]).

### In vitro motility assays

All motility assays were performed in microfluidic flow channels constructed from 22mm x 22mm glass coverslips (Menzel, Braunschweig, Germany; #1.5) and 10mm x 10mm silicon wafers having a 30nm (4nm in case of dynein gliding assays) thermally grown oxide layer (GESIM, Grosserkmannsdorf, Germany) with NESCO (Azwell Inc., Osaka, Japan) film as spacer. Typically, the flow channels have the dimensions of 10mm x 1.5mm x 100μm.

#### Kinesin-1 gliding motility assays

Flow channels (constructed of easy-cleaned [25] glass coverslips and silicon wafers with an oxide layer of 30nm) were flushed with a sequence of: (i) casein solution consisting of 0.5mg/ml casein (Sigma) in BRB80 in order to block the flow-channel surfaces (incubation time 5min), (ii) motor solution consisting of 150nM kinesin-1, 1mM MgATP (Roche), 0.2mg/ml casein, 10mM DDT (Sigma) in BRB80 in order to bind kinesin-1 proteins unspecifically to the casein surface (incubation time 5min), (iii) S-MT solution consisting of S-MTs diluted in BRB80T supplemented with 1mM MgATP, 0.2mg/ml casein, 10mM DDT (incubation time 2 min) and (iv) imaging solution consisting of 1mM MgATP, 0.2mg/ml casein, 10mM DDT, and an oxygen scavenger mixture (consisting of 40mM glucose [Sigma], 110μg/ml glucose oxidase [SERVA], 22μg/ml catalase [Sigma]) in BRB80T to remove the excess MTs. For experiments using QD-SA-B-S-MTs instead of S-MTs, 10-20pM streptavidin conjugated QDot 655 (Lifetechnologies) solution was added to the B-S-MT solution and after an incubation time of 2 min this mixture was diluted and used as stated in (iii).

#### Kinesin-8 gliding motility assays

Flow channels (constructed of dichlorodimethylsilane-coated glass coverslips [26] and silicon wafers with an oxide layer of 30nm) were flushed with a sequence of: (i) fab fragment solution consisting of 20μg/ml F(ab’)_2_ fragments (anti-mouse IgG [Fc specific] antibody developed in goat; Sigma) in PBS in order to promote anti-body binding (incubation time 5min), (ii) Pluronic F127 (Sigma, 1% in PBS) in order to block the surface from unspecific protein adsorption (incubation time 30-60min), (iii) antibody solution consisting of 0.5mg/ml anti GFP monoclonal antibodies (mouse, MPI-CBG antibody facility) in PBS in order to allow specific binding of the antibodies to the F(ab’)_2_ fragments (incubation time 10min), (iv) motor dilution buffer consisting of 112.5mM KCL, 1mM ATP (10μM ATP for the low ATP experiments), 0.1% Tween 20, 0.2mg/ml casein and 0.2mg/ml DTT in BRB80 to remove unbound antibodies, (v) motor solution consisting of 120nM Kip3-eGFP in motor dilution buffer in order to bind the Kip3 proteins specifically to the antibodies (incubation time 10min), (vi) S-MT solution consisting of S-MTs in motor dilution buffer (incubation time 5min) and (vii) imaging solution consisting of the oxygen scavenger system in motor dilution buffer to remove excess MTs. For assays with QD-SA-B-S-MTs the previously mentioned strategy (see Kinesin-1 gliding motility assays) was used in (vi).

#### Cytoplasmic dynein gliding motility assays

Flow channels (constructed of dichlorodimethylsilane-coated glass coverslips and silicon wafers with an oxide layer of 4nm) were flushed with a sequence of: (i) antibody solution consisting of 100μg/ml (20μg/ml and 10μg/ml for the lower motor density experiments) anti dynein antibody (74kDa Intermediate chains, cytoplasmic, clone 74.1, Millipore) in PBS (incubation time 5min), (ii) Pluronic F127 (Sigma, 1% in PBS) in order to block the surface from unspecific protein adsorption, (iii) motor dilution buffer consisting of 20mM KCL, 1mM ATP, 0.1% Tween 20, 0.2mg/ml casein and 0.2mg/ml DTT in BRB35 (35mM Pipes, pH 6.9, with KOH, 1mM EGTA, 1mM MgCl_2_) to wash the channel, (iv) motor solution consisting of 80nM dynein in motor dilution buffer in order to bind dynein protein to the antibodies (incubation time 5min), (v) S-MT solution consisting of S-MTs in motor dilution buffer (incubation time 5min) and (v) imaging solution with a diluted oxygen scavenger system (consisting of 40mM glucose, 55μg/ml glucose oxidase, 11μg/ml catalase) in motor dilution buffer to remove excess MTs.

### Image acquisition

Optical imaging was performed using an inverted fluorescence microscope (Zeiss Axiovert 200M, Carl Zeiss, Göttingen, Germany) with a 63x water immersion 1.2 NA objective (Zeiss) in combination with an Andor Ixon DV 897 (Andor Technology, Belfast, UK) EMCCD camera controlled by Metamorph (Molecular Devices Corporation, Sunnyvale, CA, USA) providing a pixel size of 0.254μm. A Lumen 200 metal arc lamp (Prior Scientific Instruments Ltd., Cambridge, UK) was used for epifluorescence excitation. Rhodamine-labeled S-MTs gliding on the silicon-wafer surfaces were imaged “through the solution” (i.e. on the far side of the flow channels) using a TRITC filterset (Ex 534/30x, DC BC R561, EM BL593/40, all Chroma Technology Corp., Rockingham, VT) with an exposure time of 400ms per frame. Images were recorded in time-lapse or continuous acquisition mode depending on the velocity of the MT gliding. For experiments with QD-SA-B-S-MTs, an argon-krypton mixed gas laser at 488nm (Innova 70 Spectrum, Coherent, Dieburg, Germany) was used to visualize the QDots attached to gliding MTs using a bandpass filter (Ex z488/10x, DC z488rdc, EM BL660/40, all Chroma Technology Corp., Rockingham, VT).

### Image processing

The rotational pitch of S-MTs was obtained from their kymographs (space-time intensity plots), which were generated in Metamorph. The kymographs were then analyzed with MATLAB (Mathworks, Natick, MA) using the speckle analysis method described in the Results section. QDots were tracked with FIESTA [27], an open-source tracking software based on MATLAB. To obtain the rotational pitch, QDot intensity profiles were analyzed analogous to the evaluation of the intensity profiles of individual speckles.

## Results

### Speckle analysis provides the rotational pitch of gliding MTs

Speckled MTs (S-MTs) gliding on motor-coated surfaces (Fig 1A, using the example of kinesin-1) were imaged and for every S-MT without a crossing event a kymograph was generated (typical example kymograph in Fig 1B). To extract the periodicity of the individual speckles, which exhibit periodic intensity variations over time, we used the following procedure: (i) Background correction was performed by subtracting the minimum pixel intensity along the kymograph column from all pixel intensity values in the respective column. For MTs moving significantly larger distances than the MT length over a rather inhomogeneous background the average intensity of each kymograph column (instead of the minimum) was subtracted. (ii) The kymograph was “straightened” by taking the intensity of the first horizontal line of the kymograph (which refers to the first time-frame) as a reference and cross-correlating it with the remaining lines in order to find how many pixels each time-frame needs to be shifted (Fig 1C). This pixel shift information for each time-frame also yields the instantaneous velocity. (iii) Speckles on the “straightened” kymograph were chosen by averaging the intensity along the MT over time and selecting the peaks from the averaged intensity profile (Fig 1D). In order to avoid noisy data, extremely dim speckles were ignored by selecting only peaks that were above the mean value of the averaged intensity profile. (iv) For each selected speckle, the intensity profile (averaged over a width of three pixels to overcome any inaccuracy due to pixelation of the ‘straightened’ kymograph) over time was interpolated to an intensity profile over distance using the instantaneous velocity information (especially important for MTs gliding with variable velocities). The resulting intensity profiles of the speckles showed a periodic variation in the intensity (Fig 1E and S1 Fig). (v) Performing an auto-correlation on the intensity profile for individual speckles (Fig 1F and S1 Fig) and subsequently obtaining the power spectral density (PSD) (using the smoothed periodogram method with a Hamming window) of the auto-correlation data yields the peaks corresponding to the spatial frequencies in the intensity signal for the speckles. In the example of Fig 1, the highest peak in the PSD curve for each speckle was approximately at 8μm (inset Fig 1G and S1 Fig) indicating that each speckle completed one rotation approximately every 8μm. This strategy is preferred over directly obtaining the PSD of the intensity profile of the speckles since the latter would give significance to bright speckles rather than to speckles with a pronounced periodicity. Use of the PSD of the auto-correlation removes the contribution of the intensity of the speckle and emphasizes only the periodicity of the intensity variation. (vi) The pitch of rotation of the gliding S-MT under investigation is then obtained by summation of the PSD curves for all individual speckles. The highest peak in the combined PSD curve (Fig 1G) yields the average rotational pitch of the specific S-MT. Kymographs from different S-MTs in the same experiment can then be used to determine the statistical error for the rotational pitch. Further details on the characteristics of the combined PSD curves are provided in the S1 Text.

### Kinesin-1 driven MT rotation data

**Fig 2:**
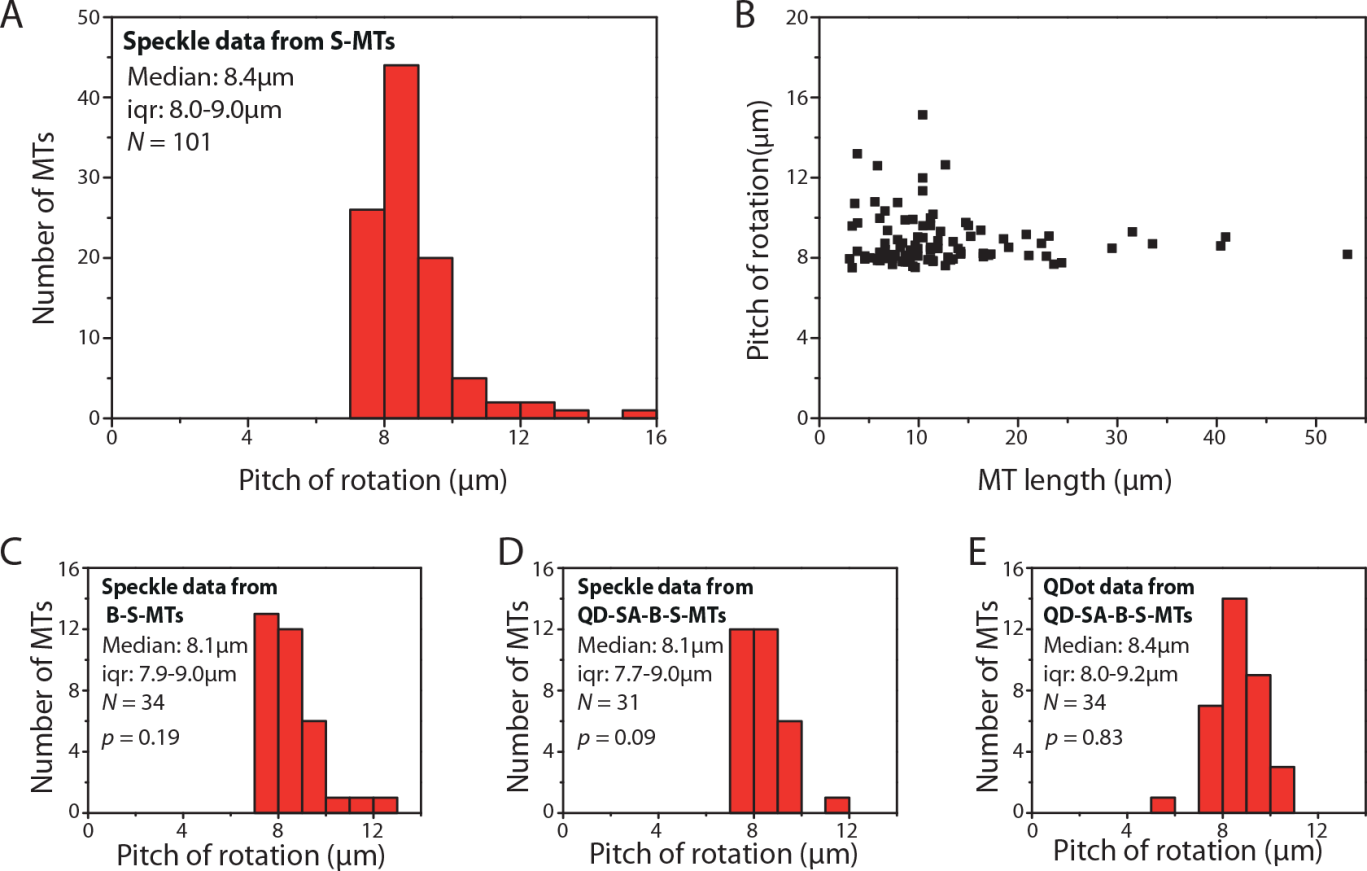
Kinesin-1 driven MT rotations. **(A)** Histogram of the rotational pitches of S-MTs. Median pitch of rotation is 8.4μm (iqr 8.0–9.0μm; *N* = 101, where *N* is the number of MTs), which is in agreement with the supertwist pitch of GMP-CPP MTs. **(B)** Variation in rotational pitch with respect to S-MT length. **(C)** Histogram of rotational pitches of B-S-MTs obtained from the speckle signal of QD-SA-B-S-MTs. Median pitch of rotation is 8.1μm (iqr 7.9–9.0μm; *N* = 34; *p* = 0.19 with respect to S-MTs, Mann-Whitney U test). **(D)** Histogram of rotational pitches obtained from the speckle signal of QD-SA-B-S-MTs. Median pitch of rotation is 8.1μm (iqr 7.7–9.0μm; *N* = 31; *p* = 0.09; *p’* = 0.56 with respect to B-S-MTs). **(E)** Histogram of rotational pitches obtained from tracking the QDots attached to QD-SA-B-S-MTs. Median pitch of rotation is 8.4μm (iqr 8.0–9.2μm; *N* = 34, where *N* is the number of QDots; *p* = 0.83).

We analyzed a total of 107 kymographs of S-MTs gliding on kinesin-1 motors (typical kymograph examples in addition to the one presented in Fig 1 are provided in S2 Fig). Only six kymographs needed to be rejected either because ‘straightening’ the kymographs was inaccurate and/or the combined PSD curve had too many significant side peaks (the same rejection criteria being applied for all following measurements). The pitch of rotation was found to be 8.4μm (median; interquartile range [iqr] 8.0–9.2μm; *N* = 101; Fig 2A). This value (i) matches previous electron microscopy data which suggested that about 95% of MTs grown in the presence of GMP-CPP are composed of 14 protofilaments with an inherent left-handed supertwist (pitch of 8.95 ± 1.36μm, calculated from the Moiré pattern periodicities [28]) and (ii) confirms earlier studies that kinesin-1 walks along single MT protofilaments [11,12]. However, the distribution of the rotational pitches was slightly skewed towards longer pitches. When plotting the rotational pitches of the individual MTs as function of MT length, we were able to attribute the longer pitches (> 10μm) to short MTs (< 12μm) exclusively (Fig 2B).

In order to cross-check our speckle-based approach to the previously employed method based on QDot tracking [25], we generated biotinylated S-MTs (B-S-MTs) to be conjugated to streptavidin-coated QDots (QD-SA). The rotational pitch of the uncoupled B-S-MTs was found to be 8.1μm (median; iqr 7.9–9.0μm; *N* = 34 out of 37 imaged B-S-MTs, Fig 2C), not significantly different from the rotational pitch of S-MTs (*p* = 0.19, Mann-Whitney U test). Likewise, the rotational pitches of QD-SA-B-S-MTs obtained by speckle analysis (8.1μm, median; iqr 7.7–9.0μm; *N* = 31 out of 35 total events; *p* = 0.09 with respect to S-MTs; Fig 2D) and QDot tracking (8.4μm, median; iqr 8.0–9.2μm; *n* = 34 out of 58, where *n* is the number of QDots tracked; *p* = 0.83 with respect to S-MTs; Fig 2E) did not show any significant differences. These results indicate that QDots have negligible impact on the torsional motion of MTs gliding on kinesin-1 coated surfaces.

### Kinesin-8 driven MT rotations

**Fig 3:**
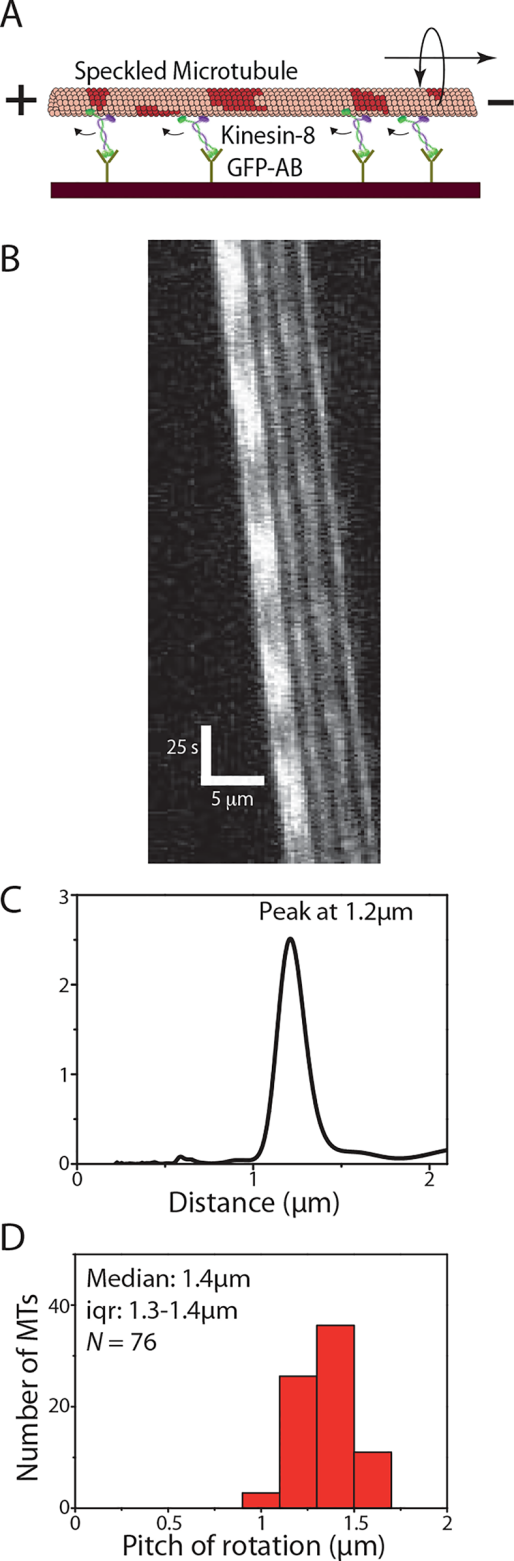
Kinesin-8 driven MT rotations. **(A)** S-MTs glide on a reflective silicon substrate coated with kinesin-8 (Kip3-eGFP) motor proteins specifically attached to the surface via GFP antibodies. **(B)** Typical kymograph of a gliding S-MT. **(C)** Combined PSD curve obtained from five speckles, with peak at about 1.2μm (data from B). **(D)** Histogram of the rotational pitches. Median pitch of rotation is 1.4μm (iqr 1.3–1.4μm; *N* = 76, where *N* is the number of MTs).

We analyzed a total of 86 kymographs of S-MTs gliding on kinesin-8 (Kip3-eGFP) motors specifically coupled to silicon-wafer surfaces by antibodies (Fig 3A). The pitch of rotation was found to be 1.4μm (median; iqr 1.3–1.4μm; *N* = 76 out of 86 imaged S-MTs; Fig 3D; typical example kymograph and evaluation in Fig 3B and 3C, S3 Fig). This value is significantly shorter than the rotational pitch on kinesin-1 coated surfaces and matches earlier results obtained by QDot tracking [13]. Again, when comparing rotation data from uncoupled B-S-MTs with rotation data obtained from QD-SA-B-S-MTs we find negligible differences in the rotational pitches (S4 Fig).

**Fig 4:**
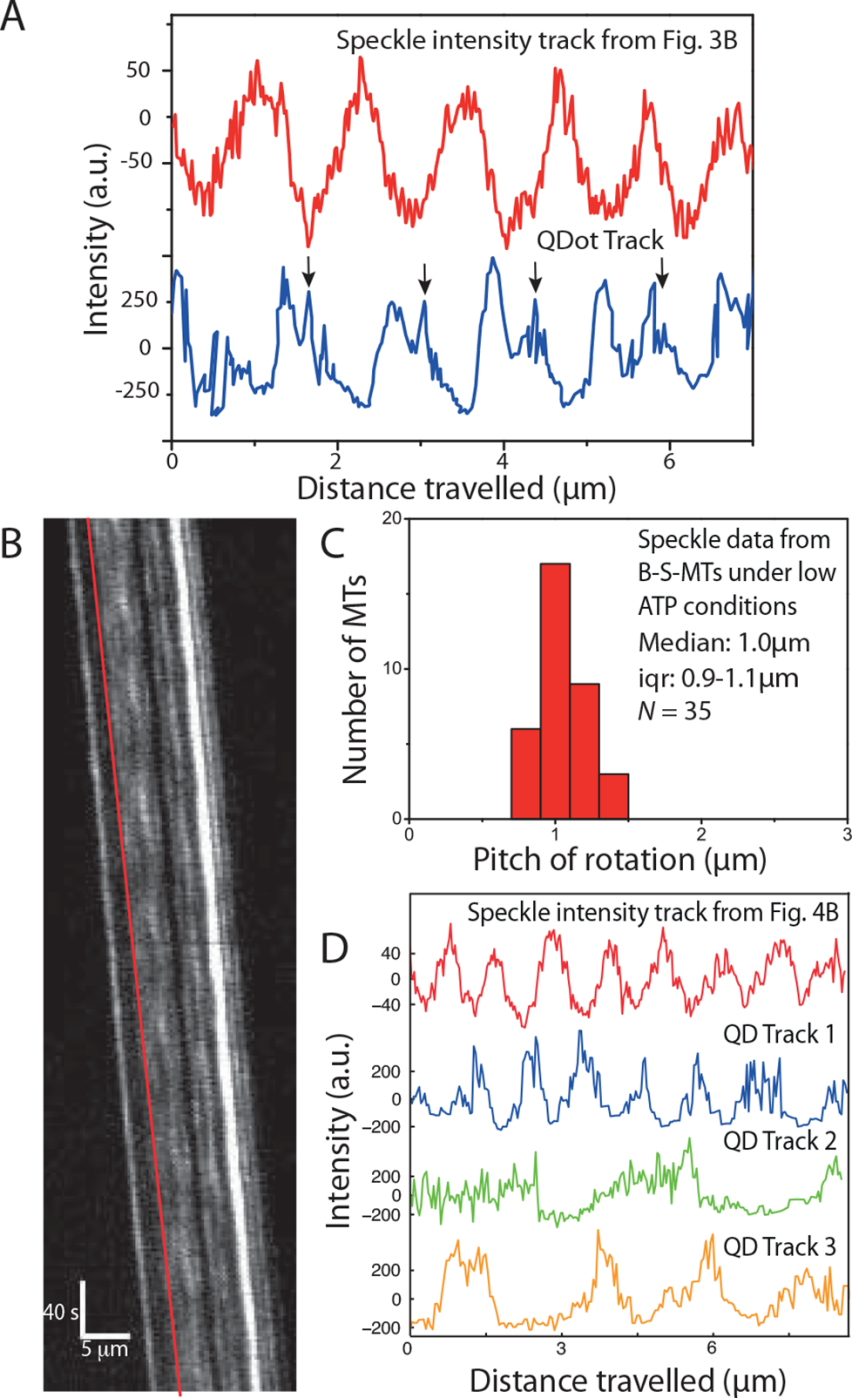
Influence of QDots coupled to MTs gliding on Kinesin-8. **(A)** Comparison of a speckle intensity profile from the S-MT shown in Fig 3B (red) and the intensity profile of a QDot on a QD-SA-B-S-MT (blue). In the speckle intensities of the S-MT, the rotational periods are symmetric and regular, in contrast to the QDot signal, which is asymmetric with regular shoulders (indicated with black arrows), suggesting that the QDot interacts with the glass substrate before it can pass between the MT and the surface. **(B)** Typical kymograph of a B-S-MT gliding on Kip3-eGFP in low (10μM) ATP conditions. **(C)** Histogram of the rotational pitches of B-S-MTs gliding on Kip3-eGFP in low ATP conditions. Median pitch of rotation is 1.0μm (iqr 0.9–1.1μm; *N* = 35, where *N* is the number of MTs). **(D)** Intensity profile of the speckle on a B-S-MT indicated by the red line in the kymograph in Fig 4B and three example QDot intensity tracks obtained from QD-SA-B-S-MTs gliding on Kip3-eGFP under low ATP conditions. The FLIC intensity data indicates that the speckle on the uncoupled B-S-MT rotates with a pitch of about 1μm while the QDots rotate differently: Track 1 (blue) ≈ 1.1μm, Track 2 (green) ≈ 3μm, Track 3 (orange) ≈ 2.3μm.

However, when plotting the QDot intensity as function of traveled distance about 40% of the intensity peaks exhibited asymmetric profiles (in contrast to the peaks obtained from tracking the speckles, Fig 4A). This observation hints towards a hindered motion of the QDots, due to surface interactions (i.e. torsional friction). In particular, the peaks in the QDot intensity profiles show shoulders (indicated by small arrows, Fig 4A) that could imply that the QDots get pressed against the surface and that the build up of sufficient torque is necessary to snap the QDots to the other side of the MTs. At reduced ATP concentration (10μM instead of 1mM), torsional friction of the QDots appeared to become dominant. Whereas uncoupled B-S-MTs rotated smoothly (see typical kymograph in Fig 4B) with a median pitch of 1.0μm (median; iqr 0.9–1.1μm; *N* = 35 out of 44 imaged B-S-MTs; Fig 4C) the QDots obstructed the torsional motion of the QD-SA-B-S-MTs with only a few MTs rotating periodically at all. In contrast to the smooth, symmetric and periodic rotations of the B-S-MTs, the intensity profiles of the few QD-SA-B-S-MTs that rotated showed variable, often hindered and asymmetric rotations (QDot tracks 1–3 in Fig 4D) without any significant reduction in the gliding velocities (gliding velocity of B-S-MTs: 13.3 ± 2.8nm/s [mean ± s.d.; *N* = 41] and gliding velocity of QD-SA-B-S-MTs: 13.5 ± 3.2 nm/s [mean ± s.d.; *N* = 21]; *p* = 0.85).

### Cytoplasmic dynein driven MT rotations

**Fig 5:**
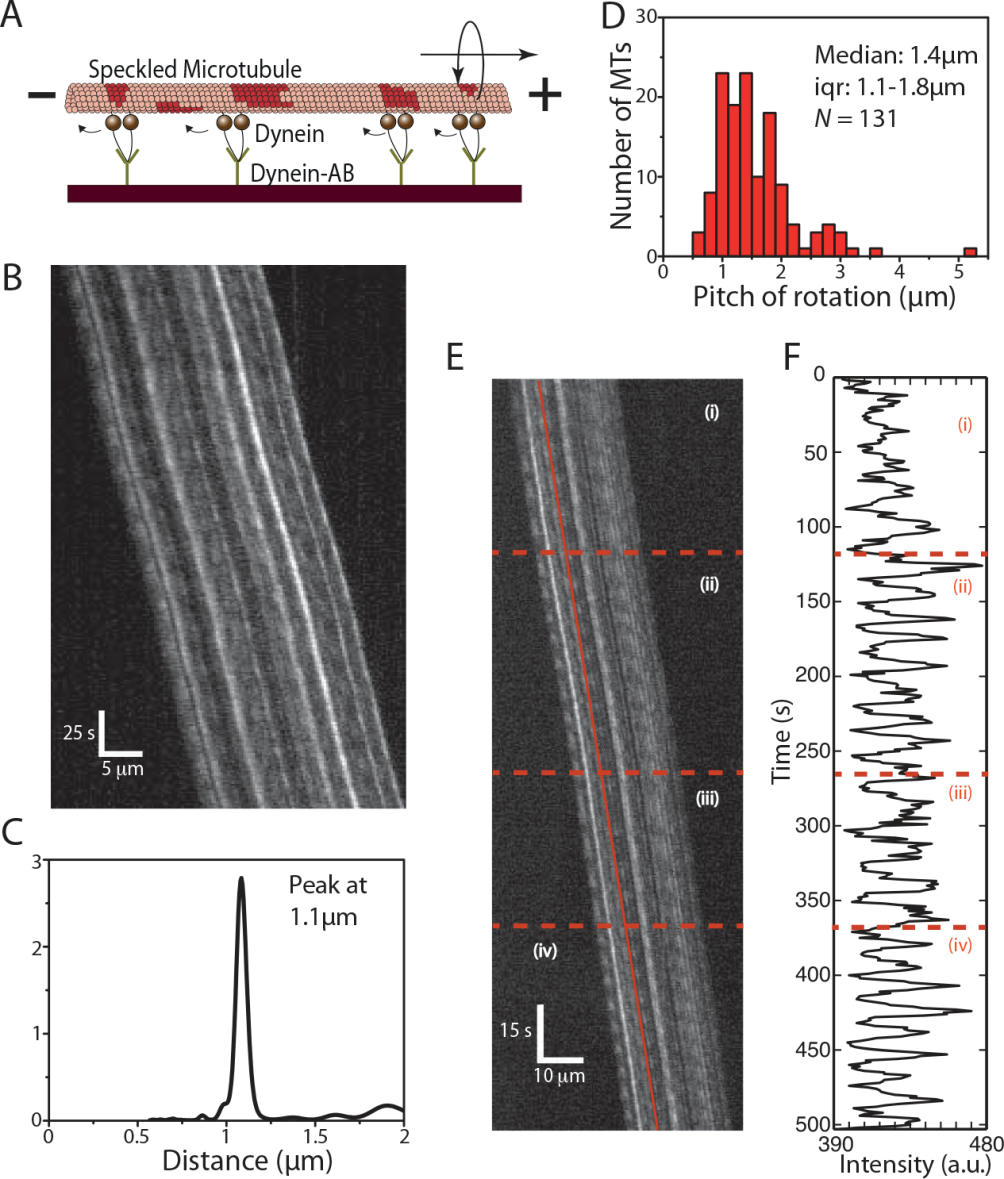
Cytoplasmic dynein driven MT rotations. **(A)** S-MTs glide on a reflective silicon substrate coated with cytoplasmic dynein motor proteins specifically attached to the surface via anti-dynein antibodies. **(B)** Typical kymograph of a gliding S-MT. **(C)** Combined PSD curve obtained from nine speckles, with peak at about 1.1μm. **(D)** Histogram of the rotational pitches, median pitch of rotation is 1.4μm (iqr 1.1–1.8μm; *N* = 131, where *N* is the number of rotation events from 75 kymographs), which indicates that the gliding MTs rotate with shorter pitches than the supertwist of the employed GMP-CPP MTs. **(E)** Typical kymograph of a gliding S-MT with varying rotational pitch. The S-MT motion is divided into four sections as can be seen in **(F)**, which shows the FLIC intensity versus time for one of the speckles on the MT lattice (indicated by the red line in E). Initially the MT had a rotational pitch of 1.1μm (i), then the rotational pitch reduced to 0.9μm (ii), followed by a switch to 1.1μm (iii), and finally, a reduction to 0.7μm (iv).

We then applied the speckle method to S-MTs gliding on *porcine* cytoplasmic dynein motors specifically coupled to silicon-wafer surfaces by anti dynein antibodies (Fig 5A). Interestingly, we found the S-MTs rotating with rotational pitches much shorter than the MT supertwist (antibody concentration = 100μg/ml, Fig 5B and 5C, S5 Fig). This observation suggests that dynein motors during collective transport switch protofilaments with a bias to a particular direction. We analyzed 118 kymographs of gliding S-MTs and observed that the rotational pitches were often not constant. 30 of the kymographs were rejected because the rotational pitches were too variable and no clear peaks were obtained in the PSD corresponding to the rotational pitch of the MT. Moreover, in a number of other kymographs, two (or more) sharp peaks were observed in the PSD indicating that the gliding MT rotated with a certain constant rotational pitch for a while before switching to another rotational pitch. In such cases the kymographs were sectioned into rotation events corresponding to different regions where the MTs rotated with fairly constant pitches for longer than four periods. The distribution in rotational pitches varied between 0.5μm and 3μm, with a median rotational pitch of 1.4μm (iqr 1.1–1.8μm; *N* = 130 rotation events from 75 kymographs, Fig 5D). A typical kymograph of a S-MT switching its rotational pitch over time is shown in Fig 5E, along with the intensity-time plot of one particular speckle shown in Fig 5F. Here, the S-MT motion could be divided into four sections: initially the rotational pitch was 1.1μm (i), reduced to 0.9μm (ii), switched back to 1.1μm (iii), and finally reduced to 0.7μm (iv).

**Fig 6:**
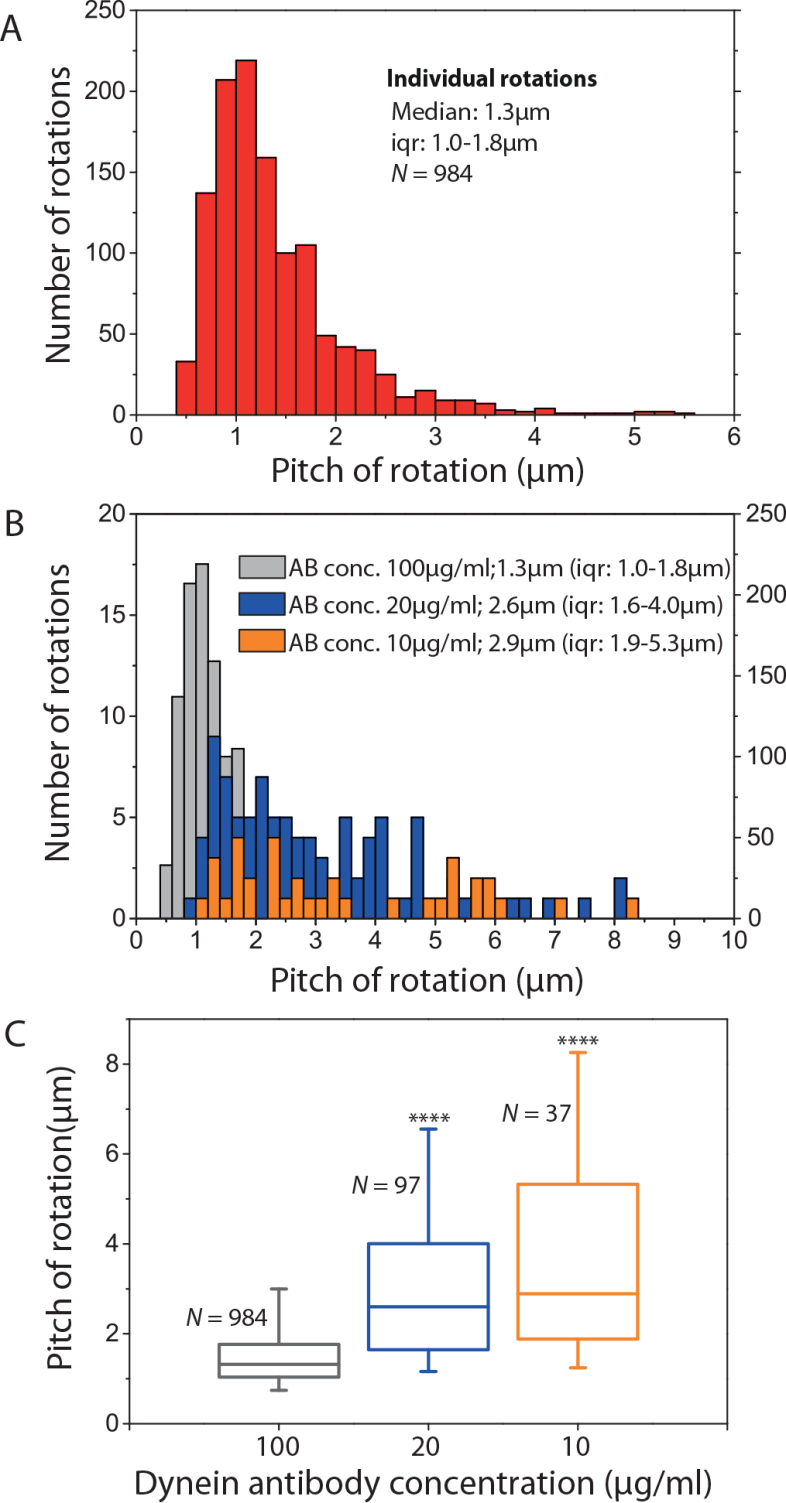
Cytoplasmic dynein driven MT rotations at different motor densities. **(A)** Histogram of individual rotations of S-MTs gliding on cytoplasmic dynein (antibody concentration 100μg/ml, analysis of the same kymographs as in Fig 5D except that the individual rotations were picked manually). **(B)** Histograms of individual rotations of S-MTs gliding on cytoplasmic dynein at different antibody concentrations and thus different motor densities (grey: antibody concentration 100μg/ml, same data as in A, y-axis on the right; blue: antibody concentration 20μg/ml, y-axis on the left; orange: antibody concentration 10μg/ml, y-axis on the left). **(C)** Box plot of the individual rotations for the three different antibody concentrations (grey: median 1.3μm, iqr 1.0–1.8μm, *N* = 984; blue: median 2.6μm, iqr 1.6–4.0μm, *N* = 97, *p* = 6.6 x 10^−26^ with respect to grey data; orange, median 2.9μm, iqr 1.9–5.3μm, *N* = 37, *p* = 1.6 x 10^−13^ with respect to grey data). **** corresponds to *p* < 0.0001.

As mentioned above, about 25% of the kymographs were rejected from the speckle analysis because our approach assigns a single rotational pitch to each gliding MT and in case of dynein the MT rotational pitches were often too irregular. Therefore, data from all kymographs were reanalyzed in more detail by choosing the clearest speckle (having the clearest intensity variation) of each S-MT and obtaining the pitches for individual rotation manually [12]. The rotational pitch obtained from the individual rotations (Fig 6A; median 1.3μm; iqr 1.0–1.8μm; *N* = 984 rotations from 105 MTs) was not significantly different from the MT rotational pitch that was analyzed automatically with the PSD (Fig 5D; *p* = 0.24).

To understand the large variability in rotational pitches of MTs gliding on cytoplasmic dynein we performed gliding assays at lower dynein surface densities by lowering the antibody concentration (10μg/ml and 20μg/ml) to which the motors are attached. At the resulting motor densities, the gliding of MTs was far less uniform (see examples of typical kymographs in S6 Fig). Also, the rotational pitches were highly variable and our speckle analysis approach did not reveal any clear peaks in the PSD except for one kymograph (S6A Fig). However, for most of the MTs (14 out of 16 MTs at antibody concentration 20μg/ml and nine out of 16 MTs at antibody concentration 10μg/ml) it was possible to obtain the individual rotational pitches by manual selection. The histograms plotted for the individual rotations (Fig 6B) reveals that the rotational pitches varied from 0.8μm to 8.5μm (at antibody concentration 20μg/ml: median 2.6μm; iqr 1.6–4.0μm; *N* = 97 rotations from 14 MTs; at antibody concentration 10μg/ml: median 2.9μm; iqr 1.9–5.3μm; *N* = 37 rotations from nine MTs). From Fig 6B and 6C it is clear that both the median of the rotational pitch as well as the variation in the pitch was significantly higher for MTs gliding on a lower density of cytoplasmic dynein.

## Discussion

We present a novel, FLIC-based method to analyze the torsional motion of MTs gliding on surfaces coated with motor proteins. Specifically, our method uses non-homogenously distributed fluorescence speckles as optical probes incorporated in the MT lattice. This way, we circumvent the previous necessity of external, often bulky probes and provide an impact-free analysis method with negligible torsional friction on MT rotations. While our method cannot readily be used to determine the handedness of the torsional motion (as it only reports on the height but not the sideward motion of the speckles), this general information can–if desired–be obtained by adding a small number of QD-SA-B-S-MTs into the same assays [12].

To benchmark our novel method, we used kinesin-1 gliding motility assays. In these experiments, we observed a rotational pitch of 8.4μm (Fig 2A), which was higher than the rotational pitch of 14 protofilament GDP-taxol MTs observed in previous works [11,29]. This implies that 14 protofilament GMPCPP MTs have a supertwist higher than the supertwist of 14 protofilament GDP taxol MTs (≈ 6μm), which has been already suggested in an earlier electron-microscopy study of MT lattice structures [28].

Unlike Nitzsche et al. [12] we did not observe MTs with rotational pitches of about 4μm (about half the average pitch). While we do occasionally identify individual speckles with a peak at half of the rotational pitch (presumably due to the collection of two or more speckles in close proximity, see S1 Text) these rare clusters did not significantly influence the combined PSD obtained from multiple speckles per S-MT and thus did not impact the rotational pitch of the S-MT. On the other hand, QDot clusters may yield similar peaks at half the rotational pitch but–due to the sparse QDot labeling of the MTs–would have a much stronger impact on the measurement.

Our results indicate that short MTs (< 12μm) have a higher variation in the measured rotational pitches (up to more than 10μm for individual S-MTs) than longer ones (Fig 2B). This variation may originate from the lower measuring accuracy since only few speckles are available for analysis on short S-MTs. An additional contribution could come from lattice defects, which may lead to the generation of S-MTs with varying protofilament numbers and reduced growth rates. However, even though we identified variations in the rotational pitches of different S-MTs, no significant differences in the rotational pitches of different parts of the same S-MT (i.e. no differences in the peak values for different speckles) were observed (S1 Table). These observations indicate (i) that there was no build-up of torsional tension in the MT lattice and (ii) that—even if MT lattice defects lead to a different supertwist in small regions of the MT—kinesin-1 motors are forced to follow the dominant supertwist of the lattice.

To investigate if the torsional motion of MTs conjugated with QDots is influenced by interactions of the QDots with the surface, we compared data from B-S-MTs gliding on kinesin-1 with QDots (QD-SA-B-S-MTs) and without QDots (B-S-MT) attached. We did not find a statistically significant difference (*p* = 0.56) in the rotational pitches, suggesting that kinesin-1 motors allow easy passage of QDots between the surface and the MT without significant hindrance of motion. For kinesin-1 gliding assays it was measured that the distance between the surface and the MT lattice is about 17nm [20]. Because the diameter of QDots (with functionalization about 25-30nm) is larger than this value, to allow the QDots to pass kinesin-1 motors may upon QDot encounter stretch (note that kinesin-1 has two hinge domains, which can readily uncoil in gliding assays [30]) or detach (note that obstacles trigger the detachment of kinesin-1 motors from the MT [31]).

When investigating the gliding of S-MTs on surfaces coated by kinesin-8 motors we found rotational pitches much shorter than the supertwist of the employed S-MTs. In consistence with previous work, this result likely originates from the capability of kinesin-8 to make stochastic side-steps to the left while moving along the MT [13]. However, in contrast to kinesin-1, coupling QDots to MTs gliding on kinesin-8 did influence their rotational pitch (Fig 4). In particular, the QDots appeared to have difficulties to pass between the surface and the MT, often getting momentarily stuck as suggested by the shoulders in Fig 4A. We see a number of possible reasons for this behavior: Kinesin-8 motors (i) are presumably rather rigid (as they do not have the hinge domains present in kinesin-1), (ii) are highly processive ([22], i.e. they have a lower detachment rate than kinesin-1), (iii) are significantly weaker than kinesin-1 [32], and (iv) are capable of switching protofilaments towards the left and right [33]. Moreover, in contrast to kinesin-1, kinesin-8 has additional MT binding sites in the motor domain [34] as well as in the neck and tail regions [35,36], tethering kinesin-8 diffusively to the MT (even in the absence of ATP [37]). Therefore, QDots can presumably only pass between the surface and the MT if sufficient torsional force is generated to force the kinesin-8 motors to detach. Then, the QDots will snap from one side of the MT to the other, resulting in the asymmetric intensity variations seen in Fig 4A. Moreover, at low ATP concentration the QDots coupled to the MT lattice partially suppressed the rotation of the QD-SA-B-S-MTs, whereas S-MTs rotated smoothly under the same conditions. This may imply that at low ATP conditions, most kinesin-8 motors are inactive and diffusively attached to the MT lattice while the few active motors that drive the MT forward do not generate sufficient torque to overcome the QDot torsional friction due to the inactive motors. This observation allows us to speculate about two things: (i) The ratio between active and inactive kinesin-8 may influence the torsional friction due to the inactive motors and/or the torsional forces that are generated by the active motors and (ii) kinesin-8 motors can sometimes be prevented from switching protofilaments towards left or even be forced to switch protofilaments towards the right. Taken together, these results show that the speckle method is superior in quantitatively detecting weak torsional motions of gliding MTs, thereby allowing the exploration of collective properties of molecular motors with a weak and/or stochastic side-stepping behavior.

Considering the aforementioned advantages of the speckle method, we used this technique to investigate the rotation of S-MTs gliding on surfaces coated with *mammalian* cytoplasmic dynein. In these experiments, we found rotations with a pitch of 1.4μm (Fig 5). At first sight, this result is not surprising, as previous work with non-processive axonemal dynein [15,16] and *yeast* cytoplasmic dynein [38] also showed torsional motion of gliding MTs and multi-motor coated beads, respectively. All these results imply a torsional component in the motor’s power stroke, which might be a characteristic feature of all dyneins. However, our results contradict single-molecule studies reporting that cytoplasmic dynein (both *yeast* [4,7,38–40] and *mammalian* [8]) steps erratically with varying step sizes and random switches to adjacent protofilaments in either direction. We hypothesize, that the torsional motion seen in multi-motor assays is not detected for single motors, because the motor domains of an individual dynein are only loosely coupled with respect to each other [39,40]. However, it is known that the collective transport by multiple dyneins can generate higher forces (at least along the longitudinal axis) [41]. This coordination could also lead to a collective torsional motion, where multiple motors are biased to side-step in the same direction.

In our experiments, MTs changed their rotational pitches while gliding on cytoplasmic dynein (Fig 5E and 5F). We hypothesize that these changes were induced by local variations of the dynein density on the surface. In support of this idea we found the rotational pitches to indeed depend on the motor density when globally varying the dynein density (Fig 6B and 6C). Smaller numbers of dynein motors propelling a MT did produce less pronounced rotations in an often quite irregular manner. These results hint towards an increasing collective effect when an increasing number of dyneins interact with the MT. How this coordination is achieved is not clear and will need further investigation.

In conclusion, the speckle method described in this paper can be applied to precisely and robustly measure the torsional motion of gliding filaments *in vitro*. The intrinsic probes introduced by the non-homogenously distributed speckles eliminate the need for bulky external probes that would potentially hinder or restrict the torsional motion, especially when investigating motor proteins with a low torque component. Additionally, potential problems associated with the crosslinking of external probes to MTs can be avoided. Application of this impact-free method to the measurements of rotations driven by kinesin-1, kinesin-8, and *mammalian* cytoplasmic dynein demonstrates the versatility of the approach, which can easily be transferred to the investigation of other MT-based motors.

## Supporting Information

S1 Fig Analysis of the individual speckles of the typical kymograph in Fig 1. **(A)** Straightened kymograph (as seen in Fig 1C) with line scans over the seven individual speckles numbered 1 to 7. The 4th speckle (line scan marked in green) is already analyzed in Fig 1. The line scans over the other speckles are marked in red. **(B)** The intensity profiles over distance, the auto-correlation of the intensity profiles and the power spectra of the auto-correlation for the speckles 1,2,3,5,6,7. The corresponding peaks for the power spectra are at 8.1μm, 7.9μm, 8.0μm, 7.8μm, 8.0μm and 7.9μm.
(EPS)

S2 Fig Four typical kymographs of S-MTs gliding on kinesin-1. The combined power spectra for the individual kymographs have their main peak at 8.0μm, 8.1μm, 7.8μm and 7.9μm. The power spectra also have a prominent peak at half distance from the main peak. This contribution is due to some speckles being in close proximity to one another. There are also peaks at higher distances that are mainly due to variable background noise in the kymographs. Vertical scale bars represent 10s and horizontal scale bars represent 5μm.
(EPS)

S3 Fig Three typical kymographs of S-MTs gliding on kinesin-8. The combined power spectra analysis of the individual kymographs yields rotational pitches of 1.5μm **(A)**, 1.2μm **(B)**, and 1.3μm **(C)**. Vertical scale bars represent 20s and horizontal scale bars represent 5μm.
(EPS)

S4 Fig Influence of QDots on kinesin-8 driven MT rotation. **(A)** Histogram of the rotational pitches of S-MTs. Median pitch of rotation is 1.4μm (iqr 1.3–1.4μm; *N* = 76, where *N* is the number of MTs). The same figure as shown in 3D. **(B)** Histogram of the rotational pitches obtained from the speckle signal of QD-SA-B-S-MTs. Median pitch of rotation is 1.3μm (iqr 1.3–1.4μm; *N* = 30; *p* = 0.49 with respect to S-MTs). **(C)** Histogram of the rotational pitches obtained from tracking the QDots attached to QD-SA-B-S-MTs. Median pitch of rotation is 1.4μm (iqr 1.3–1.5μm; *N* = 48, where *N* is the number of QDots; *p* = 0.81).
(EPS)

S5 Fig Three typical kymographs of S-MTs gliding on cytoplasmic dynein (at 100μg/ml antibody concentration). The combined power spectra analysis of the individual kymographs yields rotational pitches of 1.1μm **(A)**, 2.9μm **(B)**, and 1.3μm (in the beginning) as well as 1.1μm (in the end) **(C)**. Vertical scale bars represent 25s and horizontal scale bars represent 5μm.
(EPS)

S6 Fig Three typical kymographs of S-MTs gliding on cytoplasmic dynein (at 20μg/ml antibody concentration). The combined power spectra analysis of the individual kymographs yields a rotational pitch of 1.1μm **(A)**, but no combined peak was visible for **(B-C)**. Vertical scale bars represent 25s and horizontal scale bars represent 5μm.
(EPS)

S1 Table Comparison of results from combined power spectra and individual speckles for ten S-MTs gliding on kinesin-1. Rotational pitch obtained from the combined power spectra does not differ from the weighted mean of the rotational pitches from the individual speckles (weighted by the heights of their peaks obtained in the PSD). The variation of the rotational pitch for individual speckles is less then 0.2μm, which is much smaller than the variation of the kinesin-1 rotational pitch distribution (iqr 8.0–9.2μm). The last column specifies the number of speckles that showed clear periodicities and the total number of speckles (in brackets).
(DOCX)

S1 Text Details of the combined power spectral density (PSD) curve. (DOCX)
