# Supplementary material for: Impact-Free Measurement of Microtubule Rotations on Kinesin and Cytoplasmic-Dynein Coated Surfaces
Source: PLoS One. 2015 Sep 14;10(9):e0136920. doi: 10.1371/journal.pone.0136920 (PMC4569553; doi:10.1371/journal.pone.0136920)
Supplement: S1 Table — Rotational pitch obtained from the combined power spectra does not differ from the weighted mean of the rotational pitches from the individual speckles (weighted by the heights of their peaks obtained in the PSD). The variation of the rotational pitch for individual speckles is less then 0.2μm, which is much smaller than the variation of the kinesin-1 rotational pitch distribution (iqr 8.0–9.2μm). The last column specifies the number of speckles that showed clear periodicities and the total number of speckles (in brackets). (DOCX) [file pone.0136920.s007.docx]

**S1 Table. Comparison of results from combined power spectra and individual speckles for 10 S-MTs gliding on kinesin-1.**

|  |  |  |  |  |
| --- | --- | --- | --- | --- |
|  |  |  |  |  |
|  |  |  |  |  |
|  |  |  |  |  |
|  |  |  |  |  |
|  |  |  |  |  |
|  |  |  |  |  |
|  |  |  |  |  |
|  |  |  |  |  |
|  |  |  |  |  |
|  |  |  |  |  |

| MT # | Pitch obtained from combined PSD (μm) | Weighted mean of pitches obtained from individual speckles (μm) | Weighted S.D. of pitches obtained from individual speckles (μm) | Number of speckles |
| --- | --- | --- | --- | --- |
| 1 | 8.01 | 8.02 | 0.07 | 7(7) |
| 2 | 8.12 | 8.13 | 0.16 | 7(8) |
| 3 | 8.08 | 8.09 | 0.17 | 6(6) |
| 4 | 7.94 | 7.92 | 0.01 | 2(2) |
| 5 | 7.69 | 7.68 | 0.09 | 7(9) |
| 6 | 8.01 | 8.02 | 0.08 | 4(5) |
| 7 | 7.76 | 7.78 | 0.13 | 9(13) |
| 8 | 8.06 | 8.04 | 0.07 | 7(9) |
| 9 | 8.08 | 8.07 | 0.06 | 5(5) |
| 10 | 8.16 | 8.17 | 0.04 | 4(4) |
