# Supplementary material for: Impact-Free Measurement of Microtubule Rotations on Kinesin and Cytoplasmic-Dynein Coated Surfaces
Source: PLoS One. 2015 Sep 14;10(9):e0136920. doi: 10.1371/journal.pone.0136920 (PMC4569553; doi:10.1371/journal.pone.0136920)
Supplement: S1 Text — (DOCX) [file pone.0136920.s008.docx]

**S1 Text. Details of the combined power spectral density (PSD) curve.**

Often, as in the case of the example kymograph in Fig 1 and S1 Fig (speckle no. 3,4), the intensity variations of dimmer speckles are more pronounced than the variations of brighter speckles, which are most likely a combination of two or more speckles in close proximity. In fact, if two speckles approximately 90 degrees apart on the MT lattice are in close proximity they are detected as one speckle, but will also exhibit a pitch roughly half of the rotational pitch of the MT. Here, the PSD for that particular speckle will include two peaks, one for the true rotational pitch and one at roughly half of the true pitch, due to the superimposition of the intensity from both the speckles. In a large number of kymographs we find a small peak at half the pitch of rotation in the combined PSD curve (see kymograph 2,4 in S2 Fig), however the half peak is almost never significantly large since we average over a number of speckles.

The peaks in the PSD curve have a characteristic height and width. The peaks are higher for an auto-correlation with pronounced side peaks, implying that all the periods in the intensity profile look very similar. If the periods in the intensity profile have large internal variations due to uneven background noise, uneven illumination, bleaching or clusters of speckles, the peaks in the PSD curve will be small. The height of the peak is therefore just an indication of the quality of the kymograph. The width of the peaks in the PSD curve depends on a number of factors: (i) the spectral window chosen for fitting the raw spectral data with the smoothed periodogram, which gives an inherent width to the peaks in the PSD curve, (ii) the number of periods in the signal, with the width being smaller with larger number of periods, and (iii) the number of frequencies close to one another, due to changes in the rotational pitch of the MT while it moves.
